# Differences in the Course of Physiological Functions and in Subjective Evaluations in Connection With Listening to the Sound of a Chainsaw and to the Sounds of a Forest

**DOI:** 10.3389/fpsyg.2022.775173

**Published:** 2022-02-21

**Authors:** Petr Fiľo, Oto Janoušek

**Affiliations:** ^1^Department of Social Sciences and Sport Management, Faculty of Sports Studies, Masaryk University, Brno, Czechia; ^2^Department of Biomedical Engineering, Faculty of Electrical Engineering and Communication, Brno University of Technology, Brno, Czechia

**Keywords:** acoustic stimuli, vasoconstriction, blood volume pulse amplitude, heart rate, respiration, skin conductance level

## Abstract

We explored differences in the course of physiological functions and in the subjective evaluations in response to listening to a 7-min recording of the sound of a chainsaw and to the sounds of a forest. A Biofeedback 2000x-pert apparatus was used for continual recording of the following physiological functions in 50 examined persons: abdominal and thoracic respiration and their amplitude and frequency, electrodermal activity (skin conductance level), finger skin temperature, heart rate (pulse, blood volume pulse and blood volume pulse amplitude) and heart rate variability (HRV). The group of 25 subjects listening to the sound of a chainsaw exhibited significantly lower values of blood volume pulse amplitude, lower values in peak alpha frequency HRV and higher values in peak high-frequency HRV. In the time interval from 80 s to 209 s, in which the two groups showed the greatest differences, lower values of blood volume pulse were also recorded while listening to the sound of a chainsaw. Listening to the sound of a chainsaw is associated with a greater feeling of fatigue and higher tension, while listening to the sounds of a forest is even considered to elicit feelings of improved learning abilities. The assumption that listening to the sound of a chainsaw results in higher defense arousal was confirmed. The greater variability which is exhibited by a majority of physiological functions while listening to the forest sounds may also be an innovative finding. It seems that there are two types of arousal (sympathetic and parasympathetic) following from correlations between physiological functions and subjective assessment. Low values of blood volume pulse amplitude are especially important from the health perspective. They correspond to the amount of vasoconstriction which occurs in the endothelial dysfunction related to increased mortality, incidence of myocardial infarction, leg atherosclerosis and topically to COVID-19.

## Introduction

Exposure to sounds, particularly to those which can extend focused attention and are perceived as unpleasant, is usually associated with sustained sympathetic defensive arousal (Vila et al., 2007). If these sounds (a level of 30 dB is sufficient) are an everyday part of the individual’s living and working environment, the long-term physiological mobilisation becomes a load (Ulrich et al., 1991) capable of inducing a non-specific stressing effect (Ulrich et al., 1991; Schreiber, 2000; Stansfeld et al., 2000; Koslowsky, 2001; WHO, 2011; Hrnčíř, 2013), which leads to deepened neurotic reactions (Hrnčíř, 2013) and a higher frequency of negative emotions (Medvedev et al., 2015). The day-noise indicator did not find increased probability of myocardial infarction and hypertension below 60 dB (year’s average noise level measured in the time interval from 07:00 to 23:00), but from 55 dB to approximately 80 dB the polynomial function explains 96% of the variance (*R*^2^) in the risk of these diseases (Stansfeld et al., 2000; WHO, 2011). For example, 30% of German population was exposed to road traffic noise in 1999, the level of which ranged from 60 to 65 dB. Data were taken on the facades of their houses (Federal Environmental Agency, 2005). In this sample, 2.9% (1,629 cases per year) suffered myocardial infarction which could be attributed to this factor. Nevertheless, the study did not take into account a need of quietness (Booi and Van den Berg, 2012; Cerwén et al., 2016). Its saturation derives from a possible access to ‘quiet’ near home, which is associated with a beneficial effect on blood pressure and sleep quality (Kang et al., 2016).

However, weighted sound pressure level (SPL) has no linear relation to the physiological response of the organism, evaluation of sounds (Kantor et al., 2009) or feelings of acoustic comfort (Hall et al., 2013). The relation is strengthened mainly by acoustic characteristics of sounds, such as the frequency spectrum, amplitude, temporal envelope (Zwicker and Fastl, 1990), directivity or sharpness (proportion of high frequencies in total frequencies), roughness (ratio of burst duration to total duration) and other psychoacoustic parameters (Kang et al., 2016). The interpretation of the meaning of sounds and their similarity (Gygi et al., 2007) or their monotony, aesthetic properties (Ratcliffe et al., 2013) and context (Medvedev et al., 2015) in which they are embedded (e.g., connection with the visual stimuli, see Carles et al., 1999) is also strengthening factors.

The most unpleasant sounds are reported to be the rough, sharp and high-frequency sounds ranging from 2.5 to 5.5 kHz (Hall et al., 2013) or those at a frequency of 10 KHz (Arguelles et al., 1962).

On the other hand, non-repetitive sounds, high in uniqueness and informativeness, with an intensity less than 90 dB(A) and in the zones of low to medium frequencies, are preferred (Southworth, 1969). The varying noisiness of sounds revealed that natural sound sources (Ellermeier et al., 2001) are generally preferred over traffic and machinery noise (Carles et al., 1999; Yang and Kang, 2005; Dubois et al., 2006; Van den Bosch et al., 2018), with a higher activity of parasympathetic nervous system being recorded after exposure to them (Ulrich et al., 1991; Annerstedt et al., 2013). Birdsong and the rustling of branches were particularly positively perceived for inducing states of relaxation (Carles et al., 1999; Yang and Kang, 2005), attention restoration, stress recovery (Ratcliffe et al., 2013; Medvedev et al., 2015) and pain reduction (Diette et al., 2003). Such sounds were also part of nature-based rehabilitation which exhibited positive results in patients suffering from stress-related mental disorders (Cerwén et al., 2016).

According to Ulrich et al. (1991) and their theory of arousal, natural sounds are markedly lower stressors compared to urban sounds due to the fact that, for humans, they are not so intense and complex. While listening to them, involuntary attention and preconscious affective reactions are more involved, which can be understood by considerably longer evolutionary development and adaptation of man in the natural environment rather than in urban conditions.

However, the quality of life in urban and rural areas (acoustic environment) is not just about the above-mentioned factors affecting the auditory sensation but also with the extensive interpretation (Kang et al., 2016) and the meaning attribution (Van den Bosch et al., 2018) of sensation. In the holistic approach to sounds, a perceptual construct gets to the forefront of interest in the concept of soundscape, stored in mental representations of listeners. The form of this construct depends both on the memory, current expectations, attitudes and activities, and on the visual context, sound marks, socio-cultural background, weather, etc. Results are then culturally specific (not only physiological) responses in the culturally q*ualitative* specific ‘landscape’ of the acoustic environment (Zhang and Kang, 2007; Kang et al., 2016).

Physiological functions often mentioned in the literature associated with the research of acoustic stimuli include as: respiration (R)—thoracic respiration (ThR) and abdominal respiration (AbR), electrodermal activity (EDA), heart rate (HR; including blood volume pulse amplitude—BVPA and blood volume pulse—BVP), heart rate variability (HRV) and body temperature (BT).

During the exposure to unpleasant acoustic stimuli emotions, such as fear and anxiety usually arise (Masaoka and Homma, 1997; Kreibig et al., 2007), respiratory (frequency) rate (RR) accelerates, but tidal volume (VT) is shallower. In contrast, during the exposure to pleasant sounds, the level of agitation and anxiety decreases (Aghaie et al., 2014), RR slows down, but values of thoracic or abdominal VT apparently depend more on the degree of arousal (Gomez and Danuser, 2004) rather than on the perception of pleasure or displeasure from acoustic stimuli (Boiten, 1998). The patterns of R (VT/RR ratios) apparently differ in relation to short-term (fight or flight) or long-term (active/passive coping strategies) defense states of organism arousal (Wientjes, 1993).

EDA seems to increase when SPL exceeds 70 dB(A). However, it also depends on the frequency spectrum width of exposed sounds, on the power of their fundamental frequency (Bjork, 1986) and on the perceptual assessment (pleasantness, eventfulness and familiarity) of the sounds (Alvarsson et al., 2010). Within EDA, we analysed only its sub-component of skin conductance level (SCL), which gradually decreases while listening to pleasant sounds like relaxing music (Kuan et al., 2016) or sounds of a fountain and tweeting birds (Alvarsson et al., 2010). After exposure to stressing stimuli, the SCL decrease is more rapid while listening to birdsong and running water than while listening to unpleasant sounds, for example traffic (Alvarsson et al., 2010; Medvedev et al., 2015). Nevertheless, it depends on the type of specific emotions (Brookhuis, 2005; Kreibig et al., 2007) which must not be influenced by the parasympathetic nervous system (Kramer, 1990; Boucsein, 2005; Boucsein et al., 2012b).

BVPA is directly proportional to vascular distensibility (Dorlas and Nijboer, 1985). It can be considered as a gauge of the tone of the sympathetic nervous system (Shelley, 2007). It is activation leads to higher vasoconstriction (Lavie et al., 2000; Iani et al., 2004) and hence to lower values of BVPA (Martin et al., 2007) or BVP (Nestoriuc and Martin, 2007). Thanks to the parasympathetic nervous system of vasodilatation (e.g., respiratory sinus arrhythmia and baroreflex, see Vila et al., 2007), the BVPA values on the other hand grow (Lin et al., 2015) or exhibit a flatter curve (Kumazawa et al., 1964). Such a BVPA increase was for example observed in the exciting Dvorak music stimuli (Jellison, 1975). BVP and BVPA can positively as well as negatively correlate both with HR (Lin et al., 2015; Wesolowski et al., 2019), its variation (Awad et al., 2001; Natalini et al., 2006; Grieshaber et al., 2009) and with the systolic blood pressure (Shelley, 2007; Peper et al., 2010) and its variance (Shamir et al., 1999). Compared to R, BVPA and HR (Trotman et al., 2019) appear more sensitive (Wilson et al., 1994) as indicators of small deviations in acoustic stress (Turpin, 1986; Vila et al., 2007). Thanks to this sensitive reaction of HR to sound intensity and duration, it is possible to determine the type of reflex likely evoked in a person, whether it was the transient detection reflex, the orienting reflex, the startle reflex or the defense reflex (Turpin, 1986; Turpin et al., 1999; Vila et al., 2007).

The causes of changes in BT values will be different when the temperature on the body periphery (skin temperature) or the body core temperature (e.g., rectum and auditory passage) is concerned (Kleitman, 1963; Vinkers et al., 2013). Similarly to BVPA, finger skin temperature (FT) values measured by us relate to the sympathetic arousal (Hull et al., 1965; Charkoudian, 2003) which controls the vasoconstriction of peripheral blood vessels (Ganong, 2005; Boucsein, 2012a). During emotions, such as fear and sadness (Kreibig et al., 2007; Vinkers et al., 2010), vasoconstriction occurs resulting in the decrease of FT (Calvin and Duffy, 2007). On experiencing conducive events (Van Reekum et al., 2004) and listening to relaxing (Kuan et al., 2016) or emotional (Dibben, 2004) music, vasodilatation is higher and FT rises.

The research objective consists in seeking correlations between the act of listening to acoustic stimuli which can be considered pleasant and unpleasant, and the course of physiological functions or evaluation of the sounds. Although we chose sounds for our research that can be considered prototypical (Kang et al., 2016) and increasing the ecological validity of results, it is still a basic laboratory research, partially exploratory and partially confirmatory, which puts limits on the generalizability of the results. We see the novelty of the research mainly in the choice of physiological functions (including a detailed analysis of HRV parameters) and in the examination of differences in their variability (absolute values of standard deviations). We presume that compared with listening to forest sounds (pleasant natural acoustic stimuli), listening to the sound of a chainsaw, which can be classified in the category of unpleasant, monotonous and urban acoustic stimuli, will be associated more with the physiological manifestations of defensive arousal (Vila et al., 2007). Specifically, we presume that due to stronger contraction of muscle fibres, higher defensive arousal (sustained sympathetic arousal) will cause greater vasoconstriction that will manifest primarily in lower BVPA values (Lavie et al., 2000; Martin et al., 2007). While listening to both sounds, a similar increase can be expected in HR values (Ulrich et al., 1991; Parrot et al., 1992) or a decrease of SCL values (Gomez and Danuser, 2004; Medvedev et al., 2015) and R values, but RR values in particular will occur (Boiten, 1998; Gomez and Danuser, 2004). However, due to the monotony and unpleasantness of the sound of chainsaws, the changes can acquire higher values (Weber et al., 1980; Gomez and Danuser, 2004). The group of EPs listening to the sounds of a forest is expected to give a more positive evaluation (Carles et al., 1999; Yang and Kang, 2005) as well as more a positive and higher classification of attention restoration and stress recovery (Ratcliffe et al., 2013; Medvedev et al., 2015).

## Materials and Methods

### Research Sample

Altogether 50 voluntary examined persons (EPs) took part in our study, six females and 44 males aged 20–24 years. Gender differences were not investigated for they should not be significant (Zhang and Kang, 2007). We tried to assemble a relatively homogeneous sample of healthy EPs without baseline differences between our study groups. The EPs were students from Masaryk University, Faculty of Sports Studies. They were randomly divided into two groups by lot. In order to prevent potential carryover effects (effect of learning, habituation, monotony, etc.), each EP listened either a 7-min recording of the sound of a chainsaw (*N* = 25), or an identically long recording of the sounds of a forest (*N* = 25). Interindividual sensitiveness to noise was not registered in the EPs (Booi and Van den Berg, 2012).

### Research Design

All EPs were told emphatically not to drink alcohol for a minimum of 2 days before the measurements (Mulder and Mulder, 1987). The measurements began between 09:00 and 09:15 to ensure similar fatigue rates among the EPs, which otherwise would have been caused by different cognitive loads during the day and by the effects of biorhythms (Wever, 1979; Dawson et al., 2007). On the day of measurement, the EPs were told to follow their ordinary daily routine. Short-term illnesses and long-term health problems (allergies, anaemia, asthma, diabetes, etc.) were noted, and their possible influence was taken into account during the data analysis (Billman, 2013). The EPs were not informed about the duration or purpose of the experiment, or physiological functions to be measured. They were forbidden to talk and to move during the measurements because both could considerably affect HR, HRV (Kramer, 1990; Mulder et al., 2000; Ganong, 2005; Billman, 2013) and R behaviour (Mulder and Mulder, 1987; Mulder, 1992; Wientjes, 1993). We tried to capture or eliminate the artefacts, namely, from respiration and movement, according to recommendations for the design of biofeedback research (Boucsein, 2005; Dawson et al., 2007; Boucsein et al., 2012b). Two erroneous sections of some physiological functions from 2 EPs were discarded (*N* = 23 or *N* = 24).

Each EP was measured separately. Prior to and after the exposure to acoustic stimuli, the EPs were administered two scales (inventories) determined for self-assessment of vigilance, activity, tiredness and sleepiness—the activation scale (AS) and the visual analogue scale (VAS), which is often used in the assessment of acoustic stimuli (Hall et al., 2013). In the AS method (Monk, 1991), one of 10 polarity statements are selected on a scale from 1 to 11. In the VAS method (Monk, 1991), the respondent ticks their current mental and physical state ‘intuitively’ on the line (encodable on a scale 0–200 mm).

Mean values (categorised data) may not capture dynamic changes of physiological functions in the course of measurements, which considerably impairs their informative value, especially when the function oscillations are distinctive and occurring frequently.

Therefore, our effort was to identify ‘breaking points’, i.e., seconds or minutes in which the distinctive changes occurred in the functions. Based on our exploratory findings that are mostly in line with the literary data (Turpin, 1986; Masaoka and Homma, 1997; Boiten, 1998; Gomez and Danuser, 2004; Vila et al., 2007), we defined four time sections for the statistical comparison of differences between the average values of sounds. Time section one was a baseline (control period) and lasted 10 s before turning on the sounds. In other words, time section one might be expressed as −10–0 s. Time section two lasted from 0 to 79 s and time section three ranged from 80 s to 209 s (up to 3 ½ min), lasting 130 s (*ca*. 2 min). Time section four ranged from 210 s to 390 s (up to 6 ½ min), lasting 180 s (3 min). The last 20 s (391 s–410 s) was removed from the measurements and statistics as the course of physiological functions in this time period could have been contaminated by the arrival of researcher into the laboratory to switch off the measuring apparatus.

### Research Equipment

All measurements were carried out in an air-conditioned laboratory at the Department of Biomedical Engineering of Brno University of Technology at a constant temperature of 23°C (for the effect of ambient temperature on cognitive and physiological functions, see Wever, 1979; Dawson et al., 2007; Boucsein, 2012a) and humidity of 40–50% (Turpin et al., 1999). Measurements were taken from January to February 2020 before the COVID-19 situation. Illuminance was monitored using a light metre (luxmeter Extech HD-450) and it ranged within a relatively narrow slot of 1,000–2,500 lx in order to prevent its possible influence on physiological functions (Wever, 1979; Kramer, 1990).

Before the EP measurement was started, the SPL value was measured three times in the laboratory using a calibrated sound level metre CEM DT-173 (accuracy +/−1.4 dB, range 30 dB–130 dB, frequency range 31.5 Hz–8 kHz). The device sensor was installed horizontally above the test chair (70 cm, 80 cm and 90 cm above the seat), i.e., in the space where the heads (ears) of EPs were situated later. The SPL value was below 35 dB(A). As the laboratory did not have any special acoustic equipment (sound-proof wall cladding), we preferred using studio headphones (AKG K 240 MkII with a frequency range of 15 Hz–25 kHz and sensitivity of 104 dB) instead of speakers.

### Methodology Related to Sounds

The SPL of both sounds was measured in the laboratory in the immediate vicinity of the headphone speakers (Medvedev et al., 2015) by a sound level metre (data logger CEM DT-173). This measurement was performed again three times at the beginning of research without the participation of EPs in order to set the SPL of both sounds precisely, which was primarily derived from the highest SPL peaks (maxima; without taking into account the frequency bands).

At this initial adjustment of SPL, our effort was to preserve the naturalness of the sound on the one hand, including the natural experience of EPs with the noisiness of that particular sound (Kang, 2007), and not to exceed the difference between the SPL sound maxima by more than 10 dB(A) and not to exceed the maximum SPL level of both sounds (namely, the sound of a chainsaw) above 70 dB(A) on the other hand. We were based, for example, on the study published by Bradley and Lang (2000) who classify all noise intensity of sounds below 75 dB(A) in the category of low intensity, which should not affect the subjective evaluation of sounds unlike noise intensity above 78 dB(A) or on the study by Zhang and Kang (2007), in which the authors claim that the range of 65–70 dB(A) is not related to SPL in evaluating the acoustic comfort. We also dwelled on the findings of Kang (2007) who informs that a difference of 3 dB is on the verge of perceptible importance (significance), and only a difference of 10 dB is assessed by humans as a double increase of loudness. On the other hand, the pleasantness or unpleasantness of sounds can in some cases increase or decrease the perceived SPL value by up to 10 dB (Zhang and Kang, 2007).

Full agreement in the setting of initial SPL could not be reached due to differences between the SPL minima and maxima and due to differences in average SPL values of sounds. The sound recording of chainsaw had a greater range of SPL and contained a higher percentage noisier segments (SPL values occurring within the upper quartile of total SPL range). Concrete difference in the SPL maxima was 7 dB(A).

Values of chainsaw noisiness were min. 54.6 dB(A), max. 66.3 dB(A). The value of Q1 quartile was 62 dB(A), and the value of Q3 quartile was 65 dB(A). Values of forest noisiness were min. 48.4 dB(A), max. 59.3 dB(A), with values of quartiles Q1 and Q3 ranging from 49 to 52 dB(A).

The first audio recording contained sounds occurring naturally in the forest (rustling of tree leaves, birdsong and blowing of the wind). The second sound was the sound of a chainsaw recorded during the typically work of a forest worker branching and cross-cutting. None of the two sounds featured any exceptional single fluctuations in noisiness (‘startle’ peaks); contrariwise, the sound of chainsaw can be unambiguously characterised as monotonous and the sound of forest as naturally varying up to monotonous.

**Figure 1:**
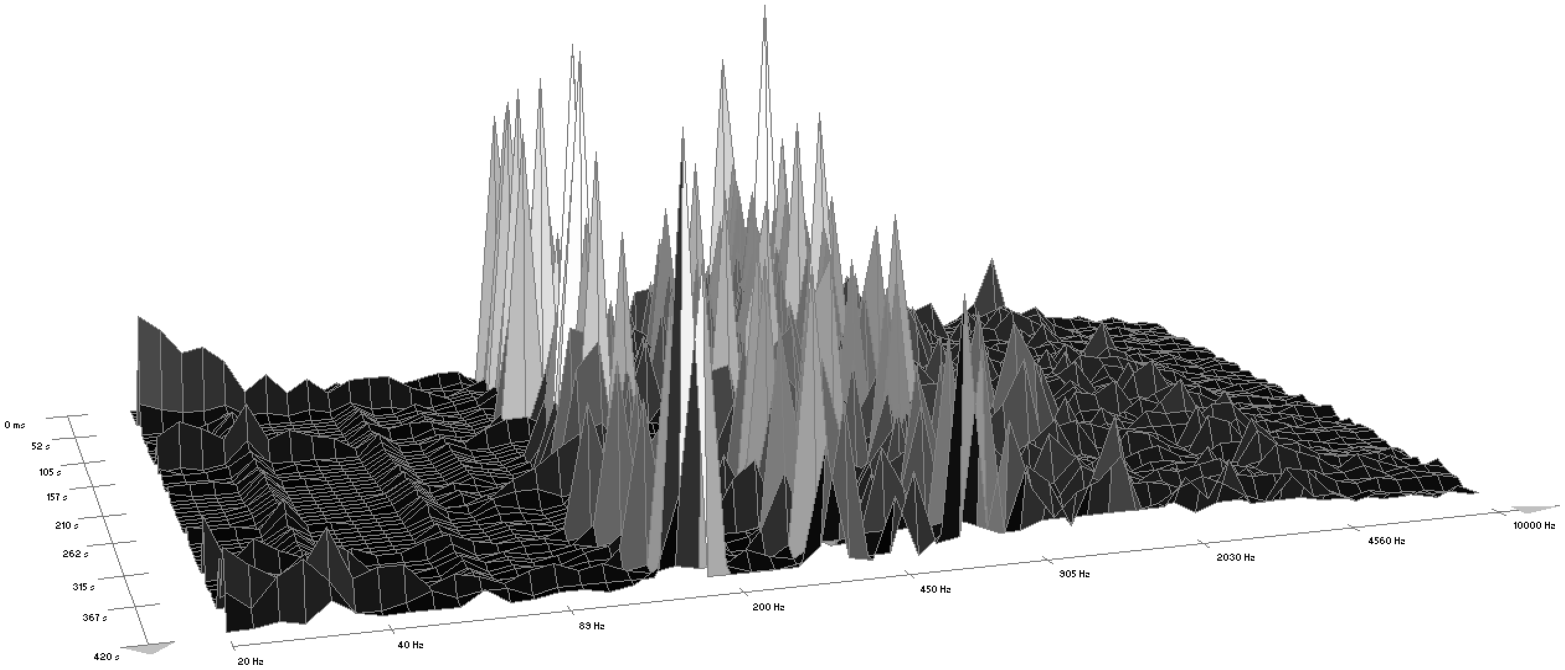
A spectrogram of the sound of a chainsaw (according to WaveLab software).

**Figure 2:**
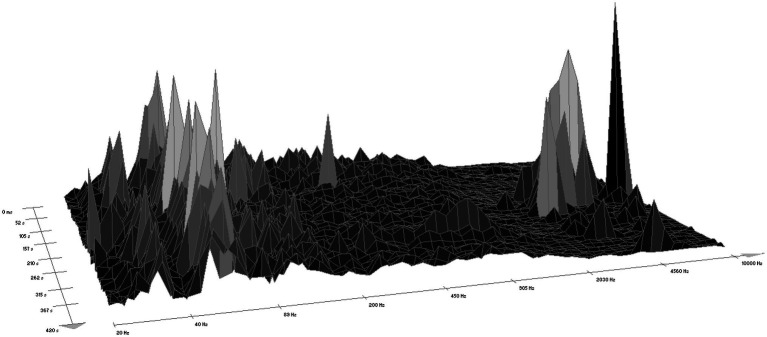
A spectrogram of the sounds of a forest (according to WaveLab software).

According to WaveLab software version 7, the sound of the chainsaw ranged within a frequency interval from 25 Hz to 10,500 Hz, most often from 110 Hz to 2,500 Hz (Figure 1). The forest sounds ranged from 25 Hz to 9,300 Hz (Figure 2), most often within two intervals, i.e., 50 Hz–200 Hz (rustling of leaves and blowing of the wind) and 2,030 Hz–9,000 Hz (birdsong).

The two sounds were recorded separately at different places (in the forest or in the open timber yard of a sawmill at a distance of about 4 m from the chainsaw operator), in 48 KHz/24 Bit quality. They were mono recorded using the Neumann U 87i variable large diaphragm microphone (maximum SPL for a total harmonic distortion of 0.5%: 117 dB (cardioid); maximum SPL for a total harmonic distortion of 0.5% with pre-attenuation: 127 dB; and frequency range: 20–20,000 Hz) and sound card Apollo Twin USB made by Universal Audio, with a max. Sampling rate 24 Bit/192 kHz. The microphone gain was set up so that recordings of both sounds ranged within the typical range of −2, 0, +2 dB. The recordings were made without any compression of the sound and were not modified later (post production sound filtering software). The microphone was wrapped in a windproof foam cover to prevent a so-called clipping or unnatural distortion of the sound, for example by husts of wind.

### Methodology Related to Biosignals

The biosignals were recorded simultaneously at the same sampling frequency of 40 Hz and the same quantifying resolution of 24 bits using a Biofeedback 2000x-pert (Schuhfried, Inc.) acquisition unit, designed for recording human biosignals under laboratory conditions. Sporadic errors occurred during testing of higher sampling frequency recordings. Though, the retained frequency shall be sufficient for measurements (Mahdiani et al., 2015; Szabolcs et al., 2019).

The R curve was recorded by means of a piezoelectric sensor with an accuracy of 1 mm, resolution of 0.2 mm and range of 10 cm. Sensors were placed on two elastic bands wrapped around the EPs’ chests over the thoracic and abdominal areas. Inspiration depth—respiration amplitude (relative tidal volume—VTR) gives the difference between the R curve maximum and minimum (mm). Respiration frequency calculates the respiration rate from the respiration curve with a range of 60 ventilations/min and a resolution of 0.02 ventilations/min.

To gauge HR parameters, the multisensor placed on the end of the left forefinger, where the sensitivity to sympathetic nervous system changes is quite large, was fitted with an infrared pulse sensor (Dorlas and Nijboer, 1985; Shelley, 2007). The multisensor was placed on the EP already before the filling of entry inventories, i.e., some 5–8 min before the start of measurements. Thanks to this, the rapid increase of body temperature and skin conductance level that appear at the beginning of measurement due to the gradual warming of the sensor to the level of finger skin temperature was eliminated. The recording of physiological functions was launched 15 s prior to the exposure to sounds. The first 5 s was cut off as a researcher was leaving the room at that time. The other 10 s served reference purposes (control period) and there was just ‘silence in the room’. We did not want to have the period longer to prevent anticipations in the perception of EPs, worries from the further course of measurements or the white coat syndrome (Pioli et al., 2018), etc. The sounds were let into the headphones of EPs by remote control from another room.

Brightness fluctuations were filtered off, amplified and recorded as blood volume pulse (BVP). These were relative changes in the blood flow, from which HR was calculated. The range of the HR parameter was 30–200 bpm (beats per minute) at a display resolution of 1 bpm. We also recorded the blood volume pulse amplitude (BVPA), whose values represent the difference between maximum and minimum BVP values during one heart cycle. BVP and BVPA are relative values (%), expressed in terms of maximum values that can be displayed and range on a scale from 0 to 100% with a resolution of 0.25%. The EDA curve carries information on the SCL and the skin conductance reflex. The skin conductance reflex is connected with the investigation of short time sections of physiological responses to the exposure of strong discontinuous stimuli, which was not our case (Boucsein, 2012a). Therefore, for the purposes of assessing the activity of sympathetic nerves, we used only SCL (μS) as a robust indicator of the sympathetic activity rate. Moreover, data of skin conductance reflex did not show any significant differences in relation to sounds.

After the measurements were complete, all data were visually inspected once again, normal values were evaluated (Dawson et al., 2007) and deviations from standard oscillations (Göbel, 2005) were examined.

For the separate analysis of HRV, which was derived from a photoplethysmographic curve, the research team used the raw data for additional calculation of R-R intervals (intervals between successive heartbeats). The R-R intervals of the BVP curve are also expressed in the range of 30–200 bpm, but with a higher resolution accuracy of 0.004 bpm. Impaired records from the BVP changes were excluded from the analysis. Peak detection in records was performed using a custom-made algorithm based on the shape analysis of filtered records. Finite impulse response low-pass filter with a cut-off frequency of 4 Hz and 30 dB ripple performed by the Chebyshev window was used to suppress noise and artefacts. Only those peaks that achieved a height of at least 10% of average peak value and a spacing higher than 375 ms were evaluated as valid peaks of pulse waves. Peak detection results were manually reviewed by the experimenter. The series of R-R intervals were derived from peak positions. HRV was analysed on the R-R interval series (Barbieri et al., 2005). Pre-processing of R-R series included the removal of ectopic intervals and R-R series detrending. Ectopic intervals differing by more than 20% were removed from the R-R series. The series of R-R intervals were detrended by wavelet packet decomposition. HRV was analysed in time, geometric and frequency domain and in non-linear domain. The heart rate variability analysis software plug-in for Matlab software was used to compute HRV parameters (Task Force of the European Society of Cardiology and the North American Society of Pacing and Electrophysiology, 1996). Standards for the evaluation of HRV unify the length of the assessed tachograph section to 5 min so that both short-term and long-term HR changes can be optimally captured for the correct quantification of which a time section of at least 2 min is needed (Task Force of the European Society of Cardiology and the North American Society of Pacing and Electrophysiology, 1996).

## Results

Firstly, we investigated differences in the average values of physiological functions for the complete interval of 7-min exposure to sounds (SPSS, version 25; Statistica, version 13.2; Matlab, version 2015b). After analysing line charts, we decided to explore the average values of standard deviations as well. A variable was created containing absolute values of standard deviations (ASD) for each N calculated according to a simple formula |SD|-average. Results of BVPASD are not presented in the paper as the data are transformed in a very similar way as BVPA. Secondly, the average values of functions and their ASD were compared to the experimental sounds in selected time sections. Four time sections, defined as a covariate, were not fixed but determined according to literary sources and similar findings from the line charts. Thirdly, based on the analysis of variance (Repeated measures or MANOVA), we further explored differences in the average values in connection with sounds and with periods. Their potential interactions were also investigated. Fourthly, we focused on the specificity in the course of the respective physiological functions and their variability (ASD). We studied time sections (‘breakpoints’) in which significant or sudden changes occurred.

### Differences in Physiological Functions From Total Time of Exposure to Sounds

Apart from peak alpha frequency HRV (Alpha), the data of other functions (means, SD) did not exhibit normal distribution, did not exceed the Kolmogorov–Smirnov boundary = *p* < 0.20; Shapiro–Wilk = *p* < 0.001. Therefore, we present both the *T*-test for independent samples and its nonparametric analogue, the Mann–Whitney test for two independent samples (Table 1). Findings of peak high-frequency HRV (Peak HF) were at a level of statistical significance; nevertheless, significant differences between the means were found in this HRV parameter in all four cases of mathematical and statistical processing: time, geometric, frequency and non-linear domain.

**Table 1:** Descriptive data and significant (bold text) differences in physiological functions ascertained by *T*-test for independent samples and the Mann–Whitney test for two independent groups.

Sounds (*N*)		Forest (25)	Chainsaw (25)	Forest (24)	Chainsaw (24)	Forest (24)	Chainsaw (24)	Forest (24)	Chainsaw (24)
Function		BVPA (%)		BVP (%)		Peak HF (−)		Alpha (−)	
Periods		1–4		3		1–4		1–4	
Independent Samples *T*-test	*t*	2.47		2.36		−2.18		2.03	
	df	43.31		34.45		39.00		46.00	
	Sig. (2-tailed)	**0.017**		**0.024**		**0.035**		**0.048**	
	SD Error Diff.	4.76		0.03		0.02		0.04	
	Power	0.69		0.62		0.62		0.48	
	Es	0.71		0.67		−0.67		0.56	
Nonparametric test	Mann–Whitney U	206.00		204.00					
	Wilcoxon W	531.00		529.00					
	Z	−2.07		−2.11					
	Sig. (2-tailed)	**0.039**		**0.035**					
Descriptive statistics	Mean	36.36	24.61	49.65	49.59	0.20	0.24	0.82	0.75
	Median	35.03	21.72	49.60	49.58	0.19	0.23	0.81	0.75
	SD	19.38	13.77	0.12	0.06	0.05	0.07	0.12	0.13
	Mean rank	29.76	21.24	29.84	21.16	20.75	28.25	28.02	20.98
	Sum of ranks	744.00	531.00	746.00	529.00	498.00	678.00	672.50	503.50

### Differences in Physiological Functions in Time Periods

As mentioned in the chapter of methodology, we defined four time sections for a partial comparison of the mean values of physiological functions. The analysis of the course of functions by means of line charts showed, for example, that the course of most of them changed dramatically in both groups of EPs from the beginning of measurement within a range of 210–240 s, from 3.5 min to 4 min. This particularly applied to AbR, SCL and HR.

**Figure 3:**
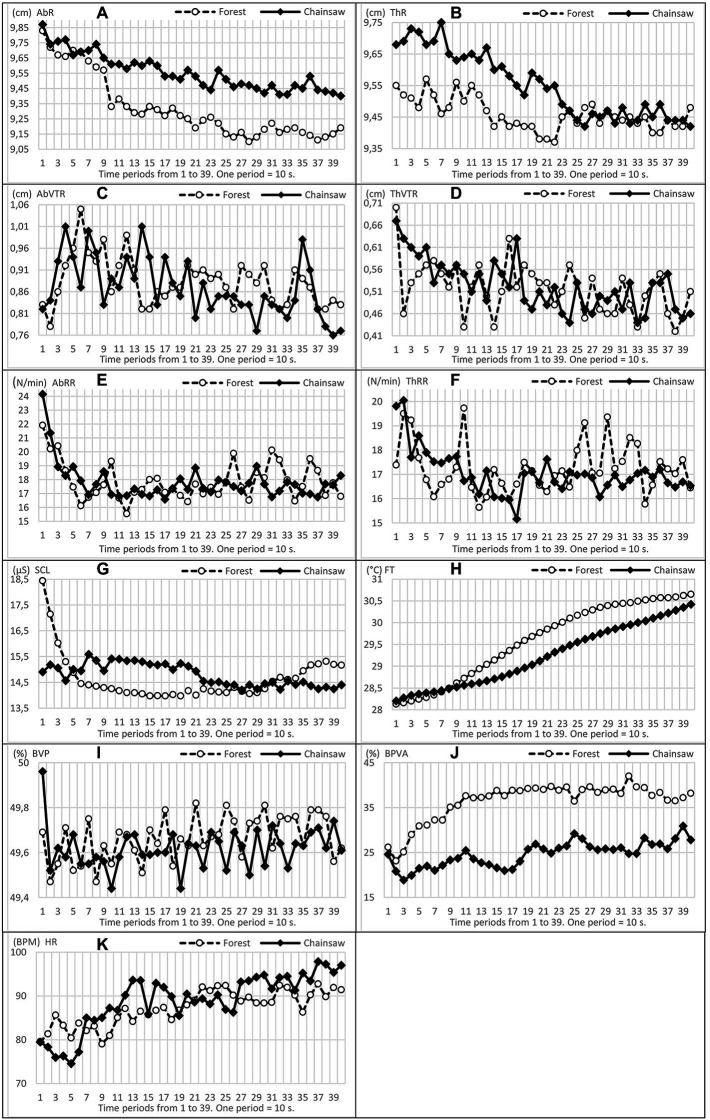
Trends in oscillations, mild and conspicuous and ascending/descending tendencies of the means of physiological functions **(A–K)**. Time axis is divided into periods from 1 to 39, where one period lasts 10 s (period 39 corresponding to the end of measurements in 390 s).

**Figure 4:**
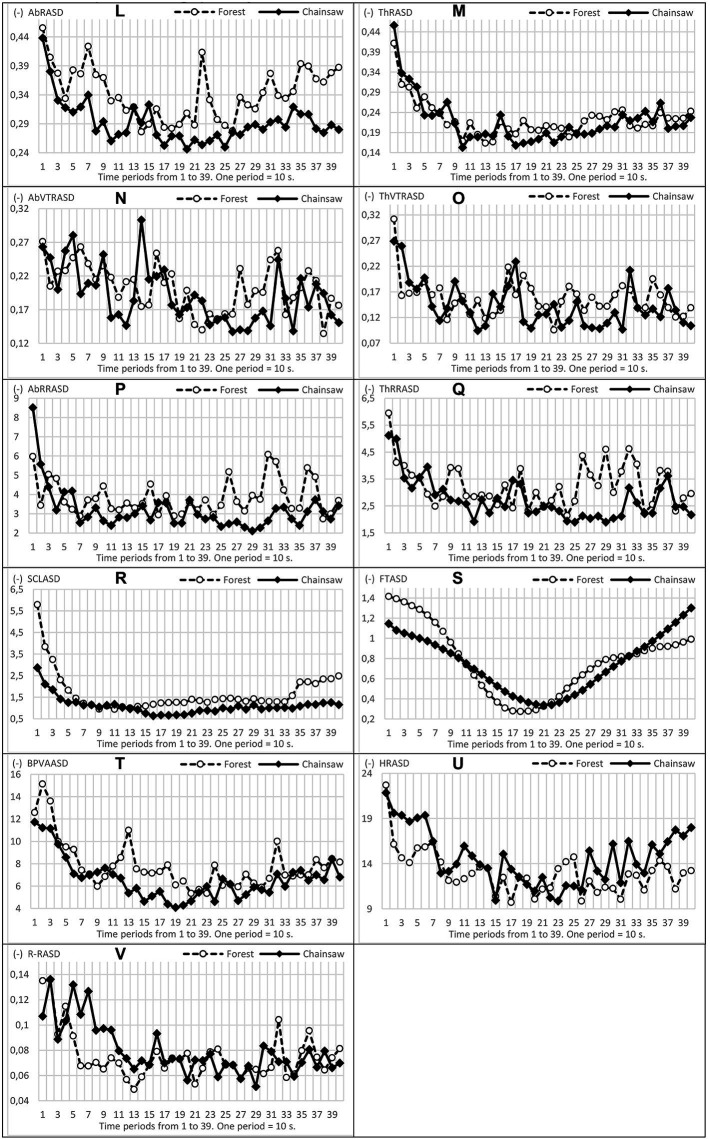
Trends in oscillations, mild and conspicuous and ascending/descending tendencies of the variability (ASD) of physiological functions **(L–V)**.

Some of the functions—BVPA, ThR and FT exhibited essential changes within an interval from 70 to 90 s. BVPA together with HR changed both after the first interval of 90 s and after the second interval of 3 ½ min. Moreover, in both groups, HR showed short-term oscillations lasting approximately 30 s or 60 s, starting at a similar time in the two groups but in the opposite direction—see Figures 3, 4. In BVPA as well as in ThR and ThVTR, the differences were most striking within a time period from 100 to 220 s.

The greatest differences in the course of the variability of functions (ASD) occurred in BVP and BVPA, again within a time section from 100 to 220 s, approximately from 2.0 to 4.0 min of measurement. In AbRR, ThRR and SCL, it was within a time interval 230–390 s, i.e., from around the fourth minute to the end of measurement, see Figure 3. In general, higher values of ASD physiological functions were, in the vast majority, recorded in EPs exposed to the forest sounds.

In the control period 1, no significant differences were recorded in association with sounds in any physiological function or its ASD. In period 2, significant differences were observed in BVPA (*T*-test: *p* < 0.019) and in period 3, a significant difference appeared also in BVP (*T*-test: *p* < 0.024; Mann–Whitney: *p* < 0.035) in addition to BVPA (*T*-test: *p* < 0.005; Mann–Whitney: *p* < 0.009). In period 4, a statistically significant difference remained again only in BVPA (*T*-test: *p* < 0.050). This shows that the greatest differences occurred while listening to sounds in period 3, both in terms of significance level and number of physiological functions.

In order to compare relations between the physiological functions and the sounds and periods, we first used the method of repeated measures. Nonetheless, after significant findings from Mauchly’s Tests of Sphericity (*p* < 0.001), we proceeded to the application of MANOVA (Table 2). However, findings from the two analyses were nearly identical. No potential interactions between the sounds and periods were found.

**Table 2:** Descriptive statistics and multivariate analysis of variance of the functions and their ASD in relation to periods and sounds (bold text).

Sounds	Forest				Chainsaw				Tests of between-subjects effects				
Periods function	1	2	3	4	1	2	3	4	Effect	***df***	Mean square	***F***	Sig.
(N/min) AbRR (*N*)	25	25	25	25	25	25	25	25	Periods	1	722.16	25.37	0.000
Mean	21.91	18.04	17.26	17.99	24.15	18.58	17.27	17.54					
SD	7.09	4.07	3.57	3.73	9.28	3.67	3.01	3.26					
(cm) ThR (*N*)	24	24	24	24	24	24	24	24	Periods	1	5.97	19.98	0.000
Mean	9.83	9.65	9.29	9.16	9.87	9.72	9.57	9.46					
SD	0.54	0.52	1.28	1.32	0.66	0.58	0.62	0.93					
(cm) ThVTR (*N*)	24	24	24	24	24	24	24	24	Periods	1	0.95	11.95	0.001
Mean	0.70	0.54	0.52	0.49	0.67	0.58	0.53	0.49					
SD	0.49	0.25	0.19	0.18	0.33	0.27	0.23	0.19					
(°C) FT (*N*)	25	25	25	25	25	25	25	25	Periods	1	103.86	5.30	0.022
Mean	28.13	28.34	29.35	3.41	28.20	28.40	28.86	29.92					
SD	4.23	4.18	4.49	4.68	4.41	4.34	4.45	4.58					
(%) BVPA (*N*)	25	25	25	25	25	25	25	25	Periods	1	1688.74	6.34	0.013
Mean	26.15	29.84	38.18	38.52	24.58	21.18	23.66	26.83	**Sounds**	**1**	**4502.56**	**16.89**	**0.000**
SD	12.56	14.85	20.34	22.97	12.00	9.81	14.11	17.73					
(BPM) HR (*N*)	25	25	25	25	25	25	25	25	Periods	1	4575.87	5.82	0.017
Mean	79.60	82.33	86.45	90.38	79.45	79.60	89.69	93.06					
SD	22.43	29.31	25.84	30.50	20.13	24.12	34.68	33.26					
(−) AbRASD (*N*)	24	24	24	24	23	23	23	23	Periods	1	0.51	12.54	0.001
Mean	0.46	0.38	0.31	0.34	0.46	0.34	0.29	0.30					
SD	0.31	0.17	0.14	0.16	0.34	0.12	0.12	0.10					
(−) ThRASD (*N*)	24	24	24	24	23	22	22	22	Periods	1	1.29	29.43	0.000
Mean	0.41	0.26	0.19	0.22	0.47	0.28	0.19	0.22					
SD	0.36	0.16	0.11	0.14	0.34	0.13	0.07	0.09					
(−) AbVTRASD (*N*)	24	24	24	24	24	24	24	24	Periods	1	0.22	8.49	0.004
Mean	0.27	0.23	0.19	0.19	0.26	0.23	0.19	0.17					
SD	0.17	0.20	0.15	0.15	0.20	0.15	0.15	0.11					
(−) ThVTRASD (*N*)	24	24	24	24	23	22	22	22	Periods	1	0.50	13.79	0.000
Mean	0.31	0.16	0.15	0.15	0.26	0.18	0.14	0.13					
SD	0.33	0.14	0.16	0.15	0.25	0.16	0.11	0.10					
(−) AbRRASD (*N*)	24	24	24	24	24	24	24	24	Periods	1	362.17	20.11	0.000
Mean	5.97	3.82	3.49	4.00	8.51	3.77	2.97	2.79					
SD	6.41	2.23	1.91	3.24	7.99	2.25	1.74	1.94					
(−) ThRRASD (*N*)	24	24	24	24	23	22	22	22	Periods	1	186.56	24.95	0.000
Mean	5.95	3.44	2.92	3.32	5.24	3.49	2.59	2.37					
SD	3.81	2.24	1.62	2.39	4.86	1.93	1.17	1.11					
(−) SCLASD (*N*)	24	24	24	24	23	23	23	23	Periods	1	207.28	17.80	0.000
Mean	5.79	2.00	1.17	1.68	2.57	1.19	0.69	0.91	**Sounds**	**1**	**77.72**	**6.68**	**0.011**
SD	7.34	2.31	1.66	2.47	3.76	1.19	1.03	1.12					
(−) FTASD (*N*)	24	24	24	24	23	23	23	23	Periods	1	9.88	18.49	0.000
Mean	1.42	1.22	0.44	0.79	1.13	0.99	0.53	0.82					
SD	0.86	0.75	0.34	0.55	0.98	0.79	0.46	0.69					
(−) BVPASD (*N*)	24	24	24	24	24	24	24	24	Periods	1	80.91	4.48	0.036
Mean	6.45	7.15	9.01	9.35	5.98	4.73	5.10	6.17	**Sounds**	**1**	**239.87**	**13.27**	**0.000**
SD	2.80	3.46	4.93	6.08	3.46	2.56	3.88	5.35					
(−) BVPAASD (*N*)	24	24	24	24	24	24	24	24	Periods	1	892.46	19.21	0.000
Mean	12.59	9.74	7.30	7.09	11.80	8.67	5.57	6.40					
SD	8.44	6.53	4.64	4.39	11.40	6.27	3.75	4.94					
(−) HRASD (*N*)	24	24	24	24	24	24	24	24	Periods	1	2430.98	11.96	0.001
Mean	22.69	14.90	11.87	12.40	21.30	16.80	12.36	13.15					
SD	22.73	12.21	8.83	8.84	21.26	13.24	9.20	8.53					
(−) R-RASD (*N*)	24	24	24	24	24	24	24	24	Periods	1	0.07	12.92	0.000
Mean	0.13	0.09	0.07	0.07	0.11	0.11	0.07	0.07					
SD	0.10	0.05	0.04	0.03	0.08	0.12	0.07	0.06					

### Courses of Physiological Functions

In addition to the basic comparison of functions and their measure of variability, we also assessed the shapes and directions (increase–decrease) of oscillations of physiological functions. Courses of all functions are presented in Figures 3 , 4. BVPA and ThRR oscillations show interesting shapes. In the case of listening to the sound of a chainsaw, ThRR exhibited a steeper decrease of values and lower variability (oscillation amplitude): particularly after the third minute, the ThRR oscillations continued in EPs listening to the forest sounds, while the oscillations slowly and relatively steadily decreased in EPs listening to the sound of the chainsaw. In the case of BVPA, its ‘synchronous’ oscillation in both groups of EPs is interesting. The values of this function were at all times markedly increasing (some peaks of oscillation) approximately at the beginning of the 3rd, 4th, 5th and 6th minutes.

### Evaluation of Sounds Before and After Their Exposure

After listening to the sounds of the forest, in the VAS method, EPs mentioned significantly higher values of their learning abilities than before listening to it (Table 3). After listening to the sound of the chainsaw, EPs stated in the AS method feeling more tired, less rested, than before exposure to the sound (Table 3).

**Table 3:** Significant differences were only recorded in nonparametric Wilcoxon’s test.

(*N* = 50)Method/inventory item/sound	Wilcoxon signed ranks test					[VAS: 0–200 mm; AS: 1–11 (−)]					*Z*	Sig. (2-tailed)	Mean rank	Sum of ranks	Mean	Median	SD	Std. error
VAS/learning/forest	pre	−2.16^b^	0.031	24.72	395.50	99.28	98.00	51.63	10.33
	post			25.14	829.50	109.40	115.00	50.96	10.19
AS/tiredness-rested/chainsaw	pre	−1.96^c^	0.050	19.57	411.00	8.68	9.00	2.21	0.44
	post			14.15	184.00	7.68	9.00	2.73	0.55

b Based on negative ranks.

c Based on positive ranks.

### Comparing the Evaluations of Sounds After Exposure

Answering the AS question ‘do you feel relaxed or do you feel tension’, EPs stated feeling significantly more relaxed—less in tension after exposure to the sounds of the forest than after having had listened to the sound of the chainsaw (Table 4). Moreover, the group exposed to the sounds of the forest gave a more homogeneous evaluation (SD 2.33 versus 3.04) on the AS scale (item relaxed-in tension) than the other group and most answers around scale points 3 and 4, indicating a high rate of relaxation (point 5.5 being the middle of the scale, indicating the answer of ‘neither – nor’). The difference is also obvious in the median value, which is around 3 in exposure to the sounds of the forest and 5 in exposure to the sound of the chainsaw.

**Table 4:** Significant differences were found both in the *T*-test for independent samples (equal variances assumed) and in its nonparametric equivalent – Mann–Whitney test for two independent groups.

(*N* = 50)	Phase	Method/inventory item	Independent samples *T*-test						
			*F*	*t*	***df***	Sig. (2-tailed)	Std. error diff.	Power	Es
	post	AS/relaxed-in tension	3.24	−2.25	48	0.029	0.77	0.60	−0.64
Sound	**Nonparametric test**				**Descriptive statistics**					Mann–Whitney U	Wilcoxon W	Z	Sig. (2-tailed)	Mean	Median	SD	Mean rank	Sum of ranks
Forest	211.00	536.00	−1.99	0.047	3.44	3.00	2.33	21.44	536.00
Chainsaw					5.16	5.00	3.04	29.56	739.00

### Correlations Between the Evaluations of Sounds and Physiological Functions

Tables 5 and 6 present Pearson correlations between the evaluations of the sounds and physiological functions in EPs pre- and post-exposure. Table 5 contains data of EPs listening to the sounds of the forest; Table 6 contains data of EPs listening to the sound of a chainsaw. In both tables, bold letters and the ^**^signs are used to highlight all the most important correlations that are significant at a level of 0.001–0.010 (two tailed). Bold italics and the ^*^sign are used to highlight correlations of medium importance (*p* < 0.028). The least important correlations that are significant at a level of 0.03–0.05 (2-tailed) are in standard font and with the ^*^sign.

**Table 5:** Pearson correlations between the (pre and post) evaluations of forest sounds and physiological functions.

Pearson corr. forest	Sig. (2-tailed)	AS pre sleepy-fresh	AS pre bad -good mood	AS pre relaxed-in tension	AS pre calm-restless	AS pre confidence-no confidence	VAS pre memory	AS post relaxed-in tension	VAS post health	VAS post no fatigue	VAS post attention	VAS post memory	VAS post thinking
AbR	*r*						−0.410^*^					−0.402^*^	
(*N* = 25)	*P* <						0.042					0.046	
AbVTR	*r*	**−0.603** ^**^											
(*N* = 25)	*P* <	**0.001**											
ThVTR	*r*			***−0.450*** ^*^									
(*N* = 24)	*P* <			***0.027***									
BVP	*r*				***−0.456*** ^*^					**0.644** ^**^	0.400^*^		
(*N* = 25)	*P* <				***0.022***					**0.001**	0.047		
BVPA	*r*							−0.407^*^	0.411^*^				
(*N* = 25)	*P* <							0.043	0.041				
PeakHF	*r*				0.434^*^								
(*N* = 24)	*P* <				0.034								
ThRASD	*r*		0.431^*^										
(*N* = 24)	*P* <		0.036										
SCLASD	*r*												**−0.543** ^**^
(*N* = 24)	*P* <												**0.006**
FTASD	*r*					0.435^*^							
(*N* = 24)	*P* <					0.034							
BVPASD	*r*								0.414^*^				
(*N* = 24)	*P* <								0.045				
HRASD	*r*												−0.415^*^
(*N* = 24)	*P* <												0.043

* Bold italics are used to highlight correlations of medium importance (*p* < 0.028). The least important correlations that are significant at a level of 0.03–0.05 (2-tailed) are in standard font.

** Highlight all the most important correlations that are significant at a level of 0.001–0.010 (two tailed).

**Table 6:** Pearson correlations between the (pre and post) evaluations of the sound of a chainsaw and physiological functions.

Pearson corr. chainsaw	Sig. (2-tailed)	AS pre calm-restless	AS pre active-inactive	VAS pre no fatigue	VAS pre thinking	VAS pre relation to exam	AS post active-inactive	AS post confid-no confid	VAS post no fatigue	VAS post attention	VAS post memory	VAS post thinking	VAS post learning
AbRR	*r*								−0.425^*^				
(*N* = 25)	*P* <								0.034				
FT	*r*				0.428^*^					***0.456*** ^*^	0.407^*^		
(*N* = 25)	*P* <				0.033					***0.022***	0.044		
AbRASD	r												−0.431^*^
(*N* = 24)	*P* <												0.036
AbVTRASD	*r*	−0.412^*^		***−0.468*** ^*^									
(*N* = 24)	*P* <	0.045		**0.021**									
AbRRASD	*r*	**−0.565** ^**^	**−0.546** ^**^										
(*N* = 24)	*P* <	**0.004**	**0.006**										
ThRRASD	*r*							−0.444^*^					
(*N* = 23)	*P* <							0.034					
SCLASD	*r*					0.409^*^	−0.423^*^					^*^	**0.519** ^**^
(*N* = 24)	*P* <					0.047	0.040					***0.023***	**0.010**

* Bold italics are used to highlight correlations of medium importance (*p* < 0.028). The least important correlations that are significant at a level of 0.03–0.05 (2-tailed) are in standard font.

** Highlight all the most important correlations that are significant at a level of 0.001–0.010 (two tailed).

Complete data from Tables 5 and 6 will be described and interpreted in detail at the end of the discussion.

## Discussion

In the literature, we very often encounter the delimitation of four responses, or reflexes, of an organism to the intensity of stimuli, transient/sustained characteristics of stimuli, response direction (latency) and habituation rate (Vila et al., 2007). These responses include transient detection reflex, orienting reflex, startle reflex and defense reflex.

Exposure to low intensity transient stimuli should elicit the transient detection reflex. An orienting response should be elicited by low intensity sustained stimuli, while the orienting reflex exhibits longer latency and faster habituation rate than the transient detection reflex. In both reflexes, HR deceleration occurs and in the case of the orienting reflex, reciprocal vasodilation-vasoconstriction in the forehead and hands has also been recorded (Vila et al., 2007). None of these manifestations of heart activity were observed in our research.

Startle response is associated with high-intensity transient stimuli; the effect has been demonstrated with stimuli of intensity of 80 dB or 100 dB and 1 s duration acoustic stimuli with a risetime of 5 ms or 30 ms (Turpin et al., 1999). The startle reflex has shorter response latency and faster habituation rate than the slower and longer defense reflex (Turpin et al., 1999; Vila et al., 2007). Stimulus intensity, however, actually had a much bigger universal effect on autonomic response components, for example HR acceleration and enhanced SCL and the late HR accelerative component amplitudes, than its duration (Turpin et al., 1999). These facts indicate that in our case the two sounds, being neither short nor noisy, should not have induced the startle reflex.

Defense response derives from high-intensity or aversive (Vila et al., 2007) sustained stimuli (the reflex was recorded, e.g., in stimuli lasting both 5 s and 30 s, see Turpin, 1986; Turpin et al., 1999). In the defense reflex, but also in the startle reflex, HR acceleration occurs immediately after the exposition to such stimuli (one fifth of a second or less). Concomitant vasoconstriction in the forehead and hands has been confirmed in the defense responses (Vila et al., 2007), which was also recorded in our research. In the defense and startle responses, the task of initial increased HR (high sympathetic reactivity) is to decrease sensory input. Such adjustment of an individual is mentioned in the proactive coping style (Koolhaas et al., 1999; Andringa and Lanser, 2013), active behavioural adjustments (Obrist, 1981) and ‘rejection hypothesis’ (Gang and Teft, 1975). However, after this first phase of HR increase, there is a considerable decrease in HR in most cases. While listening to sounds evaluated as unpleasant and highly arousing, the first HR decrease by up to 2.90 bpm had already occurred in the course of the first 6 s (Bradley and Lang, 2000; Hume and Ahtamad, 2013). These findings correspond with our research results because the initial decrease in HR by about 5 bpm was recorded only in the EPs listening to the sound of the chainsaw. Such a decrease in HR (habituation) in the defense reflex usually completely disappears within 2 to 3 min (Turpin, 1986; Vila et al., 2007). However, the most important changes in HR oscillation take place during the first 80 s, both with the noisy (109 dB) and the less noisy (79 dB) acoustic stimuli (Vila et al., 2007). This was also demonstrated in our research. While listening to the sound of a chainsaw, the habituation may be faster due to the amount of stimulus repetition and shorter time latency between the stimuli. By contrast, while listening to the sounds of a forest, the habituation may be slower thanks to higher sensory quality, the ‘natural meaning’ of the stimulus (Vila et al., 2007) and more positive emotional valence of the stimulus (Hall et al., 2013). After this phase of HR decrease, it is usual that the systematic acceleration of heart activity follows, in our case from around 80 bpm to 95 bpm, which is also confirmed by findings published by Parrot et al. (1992). In their research, male EPs were exposed for 15 min to four unpleasant sounds of maximum identical intensity of 75 dB. HR increase was most expressive in road traffic noise, which was followed by pile-driver noise and the sound of gunfire. An intermittent pink noise caused the least HR increase. These findings indirectly correspond with the results of our research. Ulrich et al. (1991) attribute the HR increase to unnatural laboratory conditions, which in our opinion might have also deepened the defense response in the case of the pleasant forest sounds.

Medvedev et al. (2015) state that in the rest phases of experiments, EPs exhibited a mild increase of HR values and a constant SCL behaviour. This combination of the course of two physiological functions is in line with our findings which primarily apply to the group of EPs listening to the forest sounds. In research conducted by Jellison (1975), EPs listened to four sounds (calming Bach and exciting Dvorak musical stimuli, white noise and silence) of intensities ranging from 69 to 76 dB, on average 72 dB. Similarly, as when listening to the forest sounds, listening to the Dvorak stimuli was accompanied by the significantly greatest increases in finger pulse volume (analogous to BVPA and sympathetic nervous system activity) when compared to the other groups. And again, similarly as in our case, although the EDA values were the lowest at listening to the exciting music, differences were not significant either in this function or in blood pressure. Two facts follow from this. BVPA could be physiological indicators in detecting changes induced by acoustic stimuli, and the forest sounds can have an exciting effect on the organism that can be compared to that of Dvorak’s music. In the study published by Ulrich et al. (1991), SCL values initially exhibited a greater increase while listening to urban, unpleasant acoustic stimuli as compared with natural, pleasant sounds. But then the two curves stagnated quite similarly as they did in our research. Interpretation of SCL behaviour is made more difficult by the fact that arousal can be associated with both pleasant and unpleasant stimuli and so EDA values can be identical in both cases. High EDA values occurring with emotional sound stimuli can, therefore, be compared only with low EDA values occurring with neutral sound stimuli (Bradley and Lang, 2000). This would mean in our case that both sounds were either neutral or emotional for the EPs, the latter being a more likely option. Different reasons for identical SCL courses can however be, at least partly and indirectly, elucidated by mutual connection with FT. During emotions, such as fear and sadness (Kreibig et al., 2007; Vinkers et al., 2010), vasoconstriction occurs and FT decreases (Calvin and Duffy, 2007). In such cases, SCL decreases with increasing BT, which we noted to a small extent in our research while listening to the chainsaw. On experiencing conducive events (Van Reekum et al., 2004) and listening to relaxing (Kuan et al., 2016) or emotional (Dibben, 2004), music vasodilatation is higher and FT rises. In such a case, SCL increases with increasing BT (Ganong, 2005; Boucsein, 2012a). This is again apparent to a small extent while listening to the forest sounds. Thus, SCL and BT values could be in substance the result of the ratio of vasoconstriction (chainsaw) and vasodilatation (forest) of blood vessels (Boucsein, 2012a).

In similar research, Masaoka and Homma (1997) explored the effect on respiration of listening to the sound of a saw mill, with a maximum recording loudness of 73 dB. Mutual comparison with our research is difficult due to the difference in audio recording length, 2 min versus our 7 min, as well as the different average loudness of exposed audio recordings (73 dB versus 64 dB). Masaoka and Homma (1997) state that compared with the 3-min control phase, the 2-min phase of exposure to unpleasant noise significantly increased both VT and RR. Nevertheless, the actual increase of values was not too great—as we found in our research. In our research, an increase of VT (AbVTR) occurred only during the first 40–60 s; otherwise, the values of both VT and RR were gradually decreasing (particularly AbRR during the first 70 s) or exhibited relatively constant behaviour. These findings could be interpreted in line with the conclusions of Gomez and Danuser (2004) or Boiten (1998). Minute ventilation increases with high arousal acoustic stimuli and decreases with low arousal stimuli. In our case, the acoustic stimuli could have become increasingly unexciting after the first 40–60 s. And finally, although Shea et al. (1987) used a similarly long 5-min audio recording in their study, their comparison of a chainsaw with white noise and listening to a storey was not optimal. Nevertheless, the increase of VT and RR values was also similarly low in their case, ranging only around 6%.

The last reflex has a form of orienting responses which are concerned with novelty and changes in stimuli. They more likely correspond to reactive coping style, passive behavioural adjustments and intake hypothesis, serving to increase sensory input (attentional process, see Turpin et al., 1999; perceptional and motivational process, see Vila et al., 2007), i.e., in situations when new stimuli should be appropriately ‘responded to’ by learning and focused attention. Unlike acoustic stimuli triggering a defense reflex or startle reflex, these stimuli must not be too intensive, above 80 dB or ranging from 60 to 100 dB, and imply threat (Stansfeld et al., 2000). Thanks to more positive evaluation and slower habituation of the forest sounds, a combination of defense reflex and orienting reflex can be assumed. The audio recording included not only monotonous natural sounds but also randomly occurring birdsong and rustling of leaves on branches which were of variable intensity. The other audio recording included mostly sounds of repetitive work with the chainsaw (branching of cut trees), so it can be ranked with the monotonous stimuli (for repetitive tasks in sawmill workers, see Johansson, 1989). Similarly as in our research, such stimuli elicit low stimulation, being over a lag of time perceived as uneventful and evaluated as highly unpleasant. If the EPs cannot have conscious control over it in order to maintain a sense of autonomy (Cerwén et al., 2016), in the sense of reduced opportunity for learning, limitation of behavioural options (Kang et al., 2016) or obstacles in meeting their long-term needs (Andringa and Lanser, 2013), they exhibit even greater defense responses associated with faster habituation (Melamed et al., 1995).

From HRV, the non-linear region can be analysed by means of tachogram (Singh et al., 2012, 2014). Our analysis of this region indicates that compared with listening to the sound of a chainsaw, listening to the sounds of the forest increased complexity in the form of peak alpha frequency parameter, which expresses the measure of self-similarity of successive heart cycles. The decreased measure of complexity is normally associated with sickness, ageing and non-normative physiology in general (Pincus and Goldberger, 1994).

Technically speaking, the effect of the parasympathetic can be detected from the ratio of high frequencies and the total HRV spectral force (Task Force of the European Society of Cardiology and the North American Society of Pacing and Electrophysiology, 1996; Floras et al., 2001). Peak high-frequency HRV is usually referred to as a reliable indicator of parasympathetic efferent activity (Pomeranz et al., 1985; Randall et al., 1991), which is significantly affected by the respiration sinus arrhythmia reflecting the immediate reduction of RR (Angelone and Coulter, 1964; Wahbeh and Oken, 2013). Although significantly higher values were measured in this parameter of HRV at listening to the sound of a chainsaw, it was only in a very narrow frequency spectrum ranging from 0.20 to 0.24 Hz. Notwithstanding that the effect of listening to the sound of a chainsaw needs not be clearly apparent on the breathing curve, a RR decrease and an increased influence of the parasympathetic could have occurred during it at least in time-limited periods.

It seems that audible safety is changing in EPs in the course of listening to the sound of a chainsaw. Thanks to subconscious subcortical processes, it should be continually increasing within the ‘fading’ of survival-driven strategies (defense reflex; Van den Bosch et al., 2018).

On the other hand, we do not dare present an unambiguous interpretation of HRV indicators because they can be distinctly influenced by breathing (Billman, 2013) or are not so sensitive to listening to sounds as the other physiological functions are (for comparison Peak HF with SCL, see Alvarsson et al., 2010).

Unlike general or appetitive arousal (Vila et al., 2007), sustained defense arousal (sustained sympathetic arousal) causes constant contraction of muscle fibres inducing vasoconstriction. The harmfulness of permanently reduced blood flow to the skin and decreased temperature of tissues (Charkoudian, 2003) is obvious. Low values of BVPA are mentioned in association with endothelial dysfunction (Kuvin et al., 2003; Lin et al., 2015), which relates to increased morbidity (Johansson, 1989) and mortality, incidence of myocardial infarction (Nilsén, 1970; Hedblad et al., 1994), leg atherosclerosis (Ögren et al., 1995; Kuvin et al., 2003) or currently with the COVID-19 disease (Varga et al., 2020). The lower BVPA values could be hypothetically associated with the more complicated cases of COVID-19. The most at-risk group from high-intensity auditory stimulation is middle-aged males (Parrot et al., 1992; Stansfeld et al., 2000; WHO, 2011) and persons exhibiting introversion, high neuroticism, low strength of excitation and strength of inhibition (Richards and Eves, 1991).

An innovative finding may be the greater variability, higher values of absolute standard deviations as well as lower values of data skewness and kurtosis, which is exhibited by a majority of physiological functions while listening to the forest sounds. As there are more of these significant differences in the individual time periods as compared with average values, and because they mostly reach higher values, examining this criterion may be beneficial for the future. In fact, the BVPA values themselves inform of pulse variability rather than of its average values. Similar results were published by Boiten (1998), who also used, among other indicators, an index of respiratory variability (coefficient of variation). Boiten found out that while watching suspenseful, tension-inducing clips from a smuggling movie, significantly less variation in expiratory time occurred as compared with the neutral, ‘vomiting’, funny etc. movie excerpts. The ‘violent’ modulation of the natural variability of the course of physiological functions itself can be thus considered a harmful phenomenon.

This fact is indirectly documented by the evaluation of the sounds. Listening to the sound of a chainsaw is associated with a greater feeling of fatigue and higher tension, while listening to the forest sounds is even considered to elicit feelings of improved memory abilities and enhanced learning capacity. The evaluation of sounds which can be related to the basic factor categories of pleasantness, eventfulness and familiarity (Axelsson et al., 2010) can be certainly induced in both sounds by the previous experience of EPs with similar examples of ‘sound marks’ because within the concept of soundscape, both can be considered as prototypical ‘iconic’ sounds (Kang et al., 2016) associated with specific contexts. Thus, the interpretation of results can be shifted from the direction of typical ‘defensive’ reflex to the concept of ‘offensive’. This puts an individual into the role of free ‘agent’ within the concept of ‘being by doing’ (Van den Bosch et al., 2018), who decides for him/herself about what he/she wants and does not want to listen to (Cerwén et al., 2016). Annoying sounds (the sound of a chainsaw) reduce in the EPs the audible indications of safety and trigger the survival (coping) mode of being, which takes them away from tranquil mind-states as well as from a possibility of ‘feeling to be present’ (Andringa and Lanser, 2013). On the other hand, it can be expected that listening to the sounds of a forest, the EPs want to be part of such a soundscape (environment) which allows them to be in tranquil mind-states and bring them into a so-called flourishing (co-creation) mode of being (Van den Bosch et al., 2018).

Discussion about the correlations between the evaluation of sounds and physiological functions is interpreted partially from our heuristic point of view and partially on the basis of similar studies (Bradley and Lang, 2000; Medvedev et al., 2015). (A similar research was conducted by Hume and Ahtamad (2013), yet the exposition to the sounds in their study lasted only 8 s). The validity of our results is also supported by the fact that all Pearson r range from 0.4 to 0.64, which are values meeting ‘the practical upper limits of correlation’ according to Guilford (1946).

A question is how much the initial subjective states of EPs can influence, before the exposure to the sounds, the subsequent values of physiological functions or how much the initial ‘tuning’ of physiological functions can influence the self-assessment before the start of measurement. According to critics of the James-Lange theory of emotions (Cannon, 1927), the influence is limited. It is also a question of how much the subjective states are associated with the physiological functions after the end of exposure to the sounds.

For some correlations, we do not have unambiguous explanations. For example, that body temperature variability (FTASD) grows with decreasing self-confidence measured before the exposure to forest sounds or that abdominal respiration increases with a lower level of memory skills felt both before and after exposure to forest sounds.

Some correlations suggest relatively logical explanations which, however, may be rather misleading without further confirmation by studies. After the exposure to both sounds, correlations appear between the increased values of physiological functions and the evaluation of mental states which reflect this activation rate. There is a correlation between higher BVP values and higher values of feelings ‘without’ fatigue and with more attention, a correlation between higher values of SCL and feelings of improvement in attention and memory, a correlation between the greater variability of SCL (SCLASD) and increasing feelings of ‘being’ active or feelings of improvement in thinking and learning or a correlation between the increasing variability of thoracic respiratory rate (ThRRASD) and feelings of greater self-confidence. Comparable results were obtained by Bradley and Lang (2000). They discovered that highly arousing sounds are associated with higher values of SCL and with an evaluated rise in the ability to recall (remember) these sounds. All these correlations could be explained by means of an inverted U in line with the Yerkes-Dodson law (Colquhoun, 1971; Kahneman, 1973). This could also be an explanation of correlations between a higher feeling of being restless and higher Peak HF values or between the feeling of improvement in thinking and an increased FT values (both feelings were detected before the exposure to both sounds). In the same sense but in the opposite direction of arousal, it would be possible to interpret correlations between the increased feelings of being sleepy, relaxed and calm and the higher BVP or both abdominal and thoracic VTR, which appear exclusively before the exposure to the sounds of a forest. Correlations between increased metabolic activity and increased temperature (Kleitman, 1963; García-García and Drucker-Colín, 1999), respiration (Wientjes, 1993), skin conductance level (Boucsein, 2012a), blood volume pulse (Nestoriuc and Martin, 2007) and Peak HF (Randall et al., 1991) are correlations proven by the Yerkes-Dodson law.

The positive influence of listening to the sounds of a forest on the human organism was documented in our research both in the form of higher BVPA levels reflecting the influence of the parasympathetic nervous system on vasodilatation (Jellison, 1975; Lin et al., 2015) and in the form of the greater variability of respiratory parameters. Benefits of this variability were corroborated by Boiten (1998) in the states of organism relaxation when watching funny movie clips. The findings can still be interpreted within the inverted U even when taking into account the defense reflex and audible safety; moreover, they support correlations of these functions with the experienced states of mind.

After the exposure to forest sounds, correlations were found between the increased values of BVPA or BVP variability (BVPASD) and the feelings of increased relaxation or improved health. Similarly, before the exposure to the sound of a chainsaw, higher values were measured in the feelings of being calm, being active or feeling without fatigue, along with higher variability values of abdominal respiratory rate (AbRRASD) or variability of VTR (AbVTRASD). Also recorded was a common increase in the feeling of a good mood, recorded before the exposure to the sounds of a forest and a variability of ThR (ThRASD).

Nevertheless, after the exposure to both sounds, correlations also occur between the faster abdominal respiratory rate (AbRR) or the higher variability of SCL (SCLASD) and AbR (AbRASD) or more frequent and bigger oscillation of HR (HRASD), and the feelings of increased fatigue or decreasing level of own thinking and learning. These facts could be explained by means of a slight amount of ‘anxious’ mental arousal (load), also known in the frame of ‘the valence–arousal model’ as the defensive motivation system with prevalent negative emotions (Bradley and Lang, 2000; Medvedev et al., 2015). Wientjes (1993) and Wilson et al. (1994) confirm that such organism setting is associated with the acceleration of RR and with shallower VT, in our case underlining the feelings of mental load and fatigue. Larger increases of the SCL (SCLASD) were in our research coupled with generally similar evaluations like in the research of Medvedev et al. (2015), concerning evaluation of soundscapes as the least pleasant and familiar and the most dominant. Though, the correlation between the changes in HR and sounds evaluation is not equivalent throughout all the mentioned studies. While these were ambiguous in Bradley and Lang (2000), Medvedev et al. (2015) detected lower changes in HR along with the increase in the area of the least pleasant and the most familiar feelings. It should be noted that the studies differ both in the choice of exposed sounds and dimensions of assessment.

The defense survival mode of being of some EPs could be hypothetically completed by the correlation between the strongly felt relation to examination, detected before the exposure to the sound of a chainsaw and the increasing variability of SCL (SCLASD). It is quite possible that the feeling of commitment is in some EPs connected with a more responsible, and hence with a more ‘stressful’ attitude to the research.

The correlations between the physiological functions and the assessment possibly confirmed the presence of two arousal types reflecting a combination of defense reflex and orienting reflex, depending on whether either dimension pleasantness or arousal dominate in the perception of sounds and the evaluation of these (Hume and Ahtamad, 2013). The first arousal, which is connected with the predominance of the parasympathetic system and with the orienting reflex, is associated with the relaxation of the organism (effect on increased attention and wellbeing, see Barber et al., 2020) and appetitive motivation (Bradley and Lang, 2000). The second arousal which causes the tension and constriction of the organism is dominated by the influence of the sympathetic system and the defense reflex (Medvedev et al., 2015). This model would explain the behaviour of the most contradictory and ambivalent physiological indicator of SCLASD, which correlates both with the positively and negatively perceived mental states and levels of EPs’ mental skills.

However, the correlations do not indicate only the linkage to exposed sounds. Sources of arousals should be also sought in the current organism setting and moods depending on the type of specific emotions (Van Reekum et al., 2004; Brookhuis, 2005; Kreibig et al., 2007; Vila et al., 2007) or on some other important elements of the perceptual construct stored in mental representations of listeners (Zhang and Kang, 2007; Kang et al., 2016).

## Conclusion

We explored differences in the course of physiological functions in response to listening to a 7-min recording of the sound of a chainsaw and to the sounds of a forest.

The assumption was confirmed that, compared to listening to forest sounds, listening to the sound of a chainsaw results in higher defense arousal. This was manifested in our study both by the lower values of blood volume pulse amplitude and finger skin temperature, increased heart rate and skin conductance level and by values of HRV parameters associated with its lower complexity (peak alpha frequency HRV) or reflecting lower respiration frequency (peak high-frequency HRV). In the time interval from 80 s to 209 s, in which the two groups showed the greatest differences, lower values of blood volume pulse were also recorded while listening to the sound of a chainsaw.

Low values of blood volume pulse amplitude refer to the presence of vasoconstriction which is associated with the incidence of endothelial dysfunction of vessels. Relevance of this disease underlines its relation to myocardial infarction, leg atherosclerosis or also to COVID-19 disease.

After listening to the sounds of a forest, significantly higher values were reported in evaluating the subjective measure of learning abilities than before listening to it. At the end of measurement, the feelings of relaxation were significantly higher than in the other group. Moreover, after having listened to the sound of the chainsaw, the EPs felt significantly more tired than before.

Findings of a great amount of differences in the variability of physiological functions between the groups could be also innovative in future clinical trials of load on the organism. This variability was, at nearly all times, significantly higher in the group listening to the forest sounds.

It seems that there are two types of arousal following from correlations between physiological functions and subjective assessment. The first sympathetic arousal (the defense reflex) could be responsible for both increased respiratory rate and greater variability of skin conductance level and more frequent and higher oscillation of heart rate, and increased feeling of fatigue as well as feelings of the decreasing level of the EPs’ own thinking and learning. The second parasympathetic arousal (the orienting reflex) could have an impact on other physiological functions and could be present in the background of all positive evaluations of explored self-dimensions.

## Data Availability Statement

The datasets presented in this study can be found in online repositories. The names of the repository/repositories and accession number(s) can be found at: [https://www.mendeley.com] repository, [doi: 10.17632/9yfddx3534.1].

## Ethics Statement

The studies involving human participants were reviewed and approved by Ethics Committee of the Department of Biomedical Engineering of the Faculty of Electrical Engineering and Communication at Brno University of Technology. The patients/participants provided their written informed consent to participate in this study.

## Author Contributions

PF: conceived and designed the experimental plan, theory and discussion, performed the experiment, statistically analyzed the data, and revised the manuscript. OJ: inspected and prepared the data including HRV frequency analysis, provided lab facilities necessary for this work. All authors have read and approved the final manuscript.

## Funding

This work was supported by a research grant of the Faculty of Sports Studies, Masaryk University, MU MUNI/51/05/2019.

## Conflict of Interest

The authors declare that the research was conducted in the absence of any commercial or financial relationships that could be construed as a potential conflict of interest.

## Publisher’s Note

All claims expressed in this article are solely those of the authors and do not necessarily represent those of their affiliated organizations, or those of the publisher, the editors and the reviewers. Any product that may be evaluated in this article, or claim that may be made by its manufacturer, is not guaranteed or endorsed by the publisher.

## Abbreviations

ASD: Absolute values of standard deviations
EP: Examined person
Es: Standardised effect
BT: Body temperature
FT: Finger skin temperature
EDA: Electrodermal activity
SCL: Skin conductance level
HR: Heart rate
BVP: Blood volume pulse
BVPA: Blood volume pulse amplitude
R-R: Intervals between successive heartbeats
HRV: Heart rate variability
HRVAS: Heart rate variability analysis software
Alpha: Peak alpha frequency HRV
Peak HF: Peak high-frequency HRV
R: Respiration
AbR: Abdominal respiration
ThR: Thoracic respiration
RR: Respiratory rate
VT: Tidal volume
VTR: Relative tidal volume change
